# Comparative Efficacy of MRI and CT in Traumatic Brain Injury: A Systematic Review

**DOI:** 10.7759/cureus.72086

**Published:** 2024-10-22

**Authors:** Muath M Dabas, Abrar D Alameri, Noor M Mohamed, Rabia Mahmood, Dong Hwi Kim, Mubushra Samreen, Ji Woo Kim, Abdullah Shehryar, Samantha Gyambrah, Adees W Bedros, Abdur Rehman, Safdar Khan

**Affiliations:** 1 Surgery, The University of Jordan, Amman, JOR; 2 Medicine, Rashid Hospital, Dubai, ARE; 3 Surgery, Dr. Sulaiman Al Habib Hospital, Dubai, ARE; 4 Accident and Emergency, Allied Hospital, Faisalabad, PAK; 5 Internal Medicine, Pusan National University, Yangsan, KOR; 6 Diagnostic Radiology, Sir Ganga Ram Hospital, Lahore, PAK; 7 Medicine, Health, and Human Sciences, Macquarie University, Sydney, AUS; 8 Internal Medicine, Allama Iqbal Medical College, Lahore, PAK; 9 Pediatrics and Child Health, Ghana Infectious Disease Center, Accra, GHA; 10 Internal Medicine, The University of Jordan, Amman, JOR; 11 Surgery, Mayo Hospital, Lahore, PAK; 12 Surgery, Services Hospital Lahore, Lahore, PAK

**Keywords:** ct, diagnostic accuracy, diffuse axonal injury, flair, gfap, mri, neuroimaging, subarachnoid hemorrhage, swi, traumatic brain injury

## Abstract

Traumatic brain injury (TBI) is a leading cause of mortality and long-term disability worldwide, and accurate imaging is essential for effective diagnosis, management, and prognosis. This systematic review evaluates the diagnostic capabilities of magnetic resonance imaging (MRI) compared to computed tomography (CT) in assessing TBI across various severities. Through a comprehensive search strategy, studies were selected that directly compared MRI and CT in TBI diagnosis, incorporating advanced MRI techniques such as susceptibility-weighted imaging and fluid-attenuated inversion recovery. The findings confirm that while CT is indispensable in acute settings for the rapid identification of life-threatening conditions such as hemorrhage and skull fractures, MRI offers superior sensitivity for detecting subtle lesions, microbleeds, and diffuse axonal injury. MRI techniques, including magnetic resonance spectroscopy, demonstrated the ability to detect metabolic changes in normal-appearing white matter, which were predictive of long-term neurological outcomes. Additionally, the integration of biomarkers, such as imaging modalities, showed the potential to improve diagnostic accuracy and reduce unnecessary CT scans. Despite the limitations related to study heterogeneity and the exclusion of non-English studies, this review underscores the complementary roles of MRI and CT in TBI management, suggesting that a combined approach can provide the most thorough assessment and improve patient outcomes. Future research should focus on large-scale trials to further refine the clinical application of these imaging modalities.

## Introduction and background

Traumatic brain injury (TBI) represents a significant medical challenge due to its complexity in diagnosis and variability in clinical outcomes. It is a leading cause of death and disability across various age groups, with pediatric populations being particularly vulnerable due to their developmental implications [1,2]. The rapid and accurate assessment of TBI is critical for effective intervention and management, which can significantly influence the long-term prognosis of patients. Imaging modalities, primarily computed tomography (CT) and magnetic resonance imaging (MRI), play pivotal roles in the initial evaluation and ongoing management of TBI [3].

CT scans are widely utilized for their speed and efficacy in detecting acute hemorrhagic events, a common occurrence in traumatic injuries. However, their ability to detect subtle changes in brain pathology or predict long-term outcomes remains limited [4]. On the other hand, MRI, including advanced techniques like T2-weighted imaging, fluid-attenuated inversion recovery (FLAIR), and susceptibility-weighted imaging (SWI), offers superior contrast resolution that helps in identifying diffuse axonal injury, microbleeds, and other parenchymal injuries that are often invisible on CT scans [5]. Recent advancements in neuroimaging techniques, such as magnetic resonance spectroscopy (MRS), further allow for the evaluation of metabolic and biochemical changes in the brain, providing deeper insights into the extent of injury and potential neuronal loss [6].

Despite these technological advancements, the selection of the most appropriate imaging modality in clinical practice involves balancing accuracy, availability, and exposure to radiation, particularly in vulnerable groups such as children. Furthermore, the introduction of biomarkers like glial fibrillary acidic protein (GFAP) and its breakdown products (GFAP-BDP) offers novel pathways for enhancing diagnostic accuracy and potentially reducing unnecessary imaging through the identification of biomolecular changes post-injury [7].

The primary objective of this systematic review is to evaluate and compare the diagnostic capabilities and clinical utility of MRI and CT in the assessment of TBI. This review aims to synthesize current evidence on how these imaging modalities contribute to the initial diagnosis, management strategies, and prognostic predictions in TBI cases. A secondary goal is to explore the integration of emerging biomarkers with traditional imaging techniques to potentially streamline the diagnostic process and improve outcome predictions. By examining a variety of studies and clinical trials, this review intends to delineate the strengths and limitations of each imaging modality across different severities and stages of TBI, thereby guiding clinical decisions and highlighting areas in need of further research. This comprehensive analysis will also consider patient-centered outcomes to ensure that the findings are relevant to improving the quality of life and long-term recovery of TBI patients.

## Review

Materials and methods

Search Strategy

The search strategy was meticulously crafted following the Preferred Reporting Items for Systematic Reviews and Meta-Analysis (PRISMA) guidelines [8] to identify studies that compare the efficacy of MRI and CT imaging techniques in the diagnosis and assessment of TBI. A comprehensive search was conducted across several major electronic databases, including PubMed, Medline, Embase, the Cochrane Library, and Google Scholar. The search covered literature from the inception of each database to the present, ensuring a thorough compilation of relevant studies.

A combination of keywords and Medical Subject Headings terms tailored to the specifics of the research question was used, such as "traumatic brain injury," "MRI," "computed tomography," "neuroimaging," and "diagnostic accuracy." Boolean operators ('AND', 'OR') were utilized to structure and refine the search queries. Examples of search strings included "MRI AND computed tomography AND traumatic brain injury," "TBI AND MRI diagnostic accuracy," and "neuroimaging AND TBI assessment." The strategy was further augmented by reviewing the reference lists of all included studies to capture any additional relevant research and by extending the search to clinical trial registries to identify unpublished or ongoing studies in this field.

Eligibility Criteria

The eligibility criteria for this systematic review were designed to rigorously select peer-reviewed research articles that assess the diagnostic effectiveness of MRI and CT in managing TBI. The inclusion criteria focused on clinical trials, randomized controlled trials, cohort studies, and meta-analyses that directly compare the diagnostic outcomes and clinical utility of these imaging modalities in human TBI patients. Eligible studies were required to be published in English and include detailed reports on the sensitivity, specificity, and prognostic assessments provided by MRI and CT, highlighting their application across varying severities of TBI.

The exclusion criteria encompassed studies that do not focus on MRI and CT comparisons, utilize animal models, or primarily investigate other imaging techniques without relevance to the core comparative analysis of MRI and CT. Non-peer-reviewed materials, such as conference abstracts and unpublished manuscripts, were also excluded to ensure the integrity of the data. Studies published in languages other than English or those lacking sufficient methodological detail to assess the outcomes of interest were similarly omitted. This approach ensured the inclusion of high-quality data pertinent to enhancing diagnostic and management strategies for TBI.

Data Extraction

The data extraction process was carefully structured to capture essential information for the systematic review comparing MRI and CT imaging in the assessment of TBI. Initially, articles identified through the search strategy were screened by two independent reviewers based on titles and abstracts to determine their relevance. Following this preliminary filter, articles classified as potentially relevant underwent a full-text review. Using a standardized form developed in Microsoft Excel (Microsoft Corporation, Redmond, CA, USA), each reviewer independently recorded data from the articles, adhering to the predefined inclusion and exclusion criteria. This form captured critical details such as the lead author’s name, publication year, study design, sample size, key findings, and noted limitations. Discrepancies between reviewers were resolved through discussion, often involving a third reviewer to ensure a rigorous and consistent evaluation of all studies. This methodical approach ensured the thorough and accurate extraction of data necessary for a detailed comparative analysis of the imaging modalities in TBI contexts.

Data Analysis and Synthesis

For the systematic review assessing the diagnostic capabilities of MRI and CT in TBI, data analysis and synthesis were conducted qualitatively due to the heterogeneity of study designs, outcomes, and imaging techniques involved. Rather than conducting a meta-analysis, a narrative synthesis approach was employed to integrate and interpret the findings across the included studies. This method allowed for the exploration and contextualization of the specific diagnostic advantages and limitations of each imaging modality as reported in the studies. The extracted data were organized thematically to identify and discuss common patterns, discrepancies, and insights regarding the effectiveness and utility of MRI versus CT in different TBI scenarios. This narrative synthesis facilitated a deeper understanding of the nuanced roles these imaging tools play in clinical settings while also highlighting areas requiring further investigation, thereby providing a comprehensive overview of the current knowledge and gaps within the field.

Results

Study Selection Process

**Figure 1:**
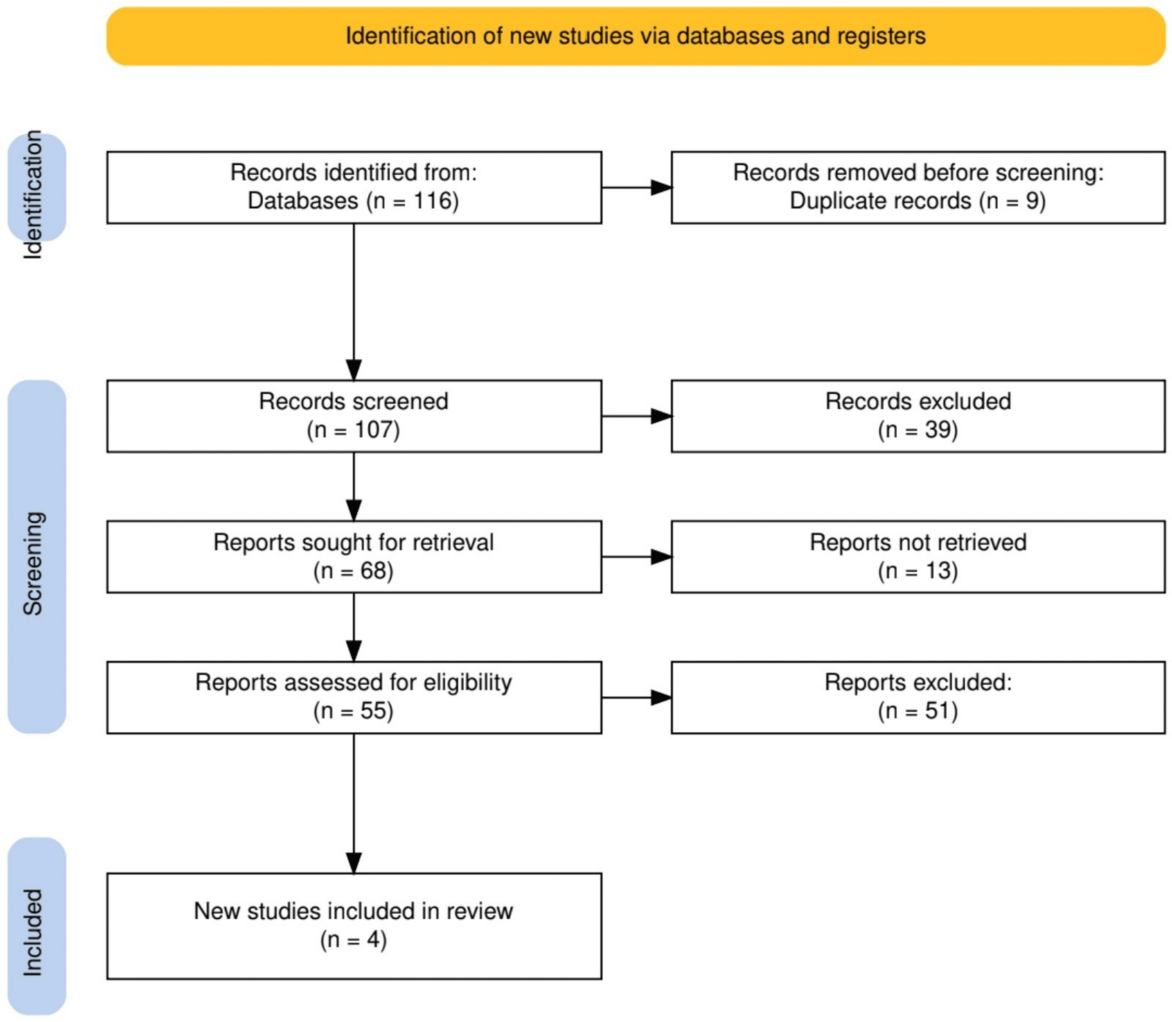
PRISMA flowchart representing the study selection process PRISMA: Preferred Reporting Items for Systematic Reviews and Meta-Analyses

The study selection process followed a systematic approach to identify and include relevant studies for the review, as shown in Figure 1. Initially, a total of 116 records were identified from database searches, with nine duplicates being removed prior to the screening. The remaining 107 records were then screened based on titles and abstracts, of which 39 were excluded due to irrelevance. For the 68 reports deemed potentially eligible, full-text retrieval was sought, but 13 reports were not retrieved. After assessing the remaining 55 full-text reports for eligibility, 51 were excluded for not meeting the predefined inclusion criteria. Ultimately, four new studies were included in the systematic review, reflecting a focused and rigorous selection process to ensure the inclusion of high-quality, relevant research.

Characteristics of the Selected Studies

The selected studies encompass a diverse range of research methodologies, populations, and imaging techniques focused on TBI. These studies include multi-center cohort analyses, comparisons of neuroimaging modalities, and MRS evaluations. The sample sizes varied from 19 to 215 patients, covering a broad spectrum of TBI severities and patient demographics, including pediatric cases. Key findings highlighted the differential effectiveness of MRI and CT in detecting specific injury types, with MRI demonstrating superior sensitivity in identifying subtle lesions and long-term brain damage, while CT remained essential for acute assessments. Table 1 summarizes the key characteristics and clinical relevance of each study.

**Table 1:** Key studies discussed in the systematic review TBI: traumatic brain injury, SWI: susceptibility-weighted imaging, CT: computed tomography, SAH: subarachnoid hemorrhage, MRS: magnetic resonance spectroscopy, GFAP-BDP: glial fibrillary acidic protein and its breakdown products, AUC: area under the curve, FLAIR: fluid attenuated inversion recovery, MRI: magnetic resonance imaging

Lead author	Study type	Population	Sample size	Key findings	Clinical relevance
McMahon et al., 2015 [9]	Multi-center, prospective, cohort study	Patients aged 16-93 years presenting with suspected TBI	215 patients	GFAP-BDP demonstrated very good predictive ability for intracranial injury on CT (AUC=0.87). It showed significant discrimination of injury severity with an odds ratio of 1.45 (95% CI, 1.29-1.64). Using GFAP-BDP could reduce unnecessary CT scans by 12-30% when used alongside other clinical information.	GFAP-BDP can help in establishing or excluding the diagnosis of radiographically apparent intracranial injury across the spectrum of TBI and may reduce unnecessary imaging procedures.
Sigmund et al., 2007 [10]	Comparison of neuroimaging modalities in pediatric TBI	40 children with TBI	40 patients	T2, FLAIR, and SWI MRI sequences showed no significant difference in lesion volume between normal and mild outcome groups but did indicate significant differences between normal and poor and between mild and poor outcome groups. CT revealed no significant differences in lesion volume between any groups.	MRI techniques (T2, FLAIR, SWI) are more effective than CT in assessing the severity of brain injuries in pediatric patients and predicting long-term outcomes. CT remains crucial for acute assessments to determine the need for neurosurgical interventions.
Wu et al., 2010 [11]	Comparison of SWI and CT for evaluating traumatic SAH	20 acute TBI patients with SAH identified by CT	20 patients	SWI identified 55 areas of SAH, matching CT findings, and detected 13 additional areas not seen on CT. CT identified 10 areas not visible on SWI. SWI was particularly effective in highlighting the unique morphology and signal intensity of SAH, showing five more cases of intraventricular hemorrhage than CT.	SWI provides complementary information to CT in detecting small amounts of SAH and intraventricular hemorrhage, demonstrating its potential for detailed assessment in TBI cases. SWI's sensitivity to blood products offers a nuanced view of hemorrhage that can enhance diagnostic accuracy and inform clinical decisions. SWI can be a valuable addition to standard CT imaging in the evaluation of traumatic subarachnoid hemorrhage.
Garnett et al., 2000 [12]	MRS study comparing cellular damage in normal-appearing white matter in head-injured patients	19 head-injured patients who were clinically stable post-TBI	19 patients	The study found a reduced N-acetylaspartate/creatine ratio and an increased choline/creatine ratio in the normal-appearing white matter of TBI patients compared to controls, correlating with injury severity. Even mildly injured patients showed significant alterations in these biomarkers.	Proton MRS provides valuable insights into the metabolic changes in white matter following TBI that are not visible on conventional MRI, offering a potential tool for early detection of cellular damage that could predict long-term neurological outcomes. This technique can be particularly useful in assessing patients with mild to moderate injuries who may experience delayed symptoms.

Discussion

The review highlights key differences in the diagnostic performance of MRI and CT in assessing TBI. Across several studies, MRI consistently demonstrated superior sensitivity in detecting subtle injuries and long-term damage. For instance, Sigmund et al. [10] found that advanced MRI sequences, such as T2, FLAIR, and SWI, provided a more accurate assessment of injury severity and predicted long-term outcomes in pediatric TBI, particularly when compared to CT. Similarly, Wu et al. [11] showed that SWI detected additional areas of subarachnoid hemorrhage (SAH) and intraventricular hemorrhage that were missed by CT. While CT was effective in acute injury assessments, MRI proved to be more sensitive in identifying microbleeds and metabolic changes in brain tissue, as evidenced by Garnett et al. [12], who utilized MRS to detect cellular damage in normal-appearing white matter.

The ability of MRI to detect subtle lesions and metabolic changes likely stems from its higher resolution and specialized imaging sequences like SWI and FLAIR, which outperform CT in identifying microbleeds and diffuse axonal injury, critical for long-term prognostic evaluation [13]. This is particularly important for mild and moderate TBI, where conventional CT might fail to show abnormalities, as demonstrated by Garnett et al. [12]. However, CT remains essential in acute settings due to its speed and efficiency in detecting life-threatening conditions such as acute hemorrhages, as noted in McMahon et al. [9]. The inclusion of GFAP-BDP biomarker studies further supports that combining CT with adjunct diagnostic tools can reduce unnecessary scans and improve decision-making. Thus, while MRI provides superior long-term diagnostic value, CT retains a crucial role in the rapid triage and management of acute TBI.

The findings of this review are consistent with the established understanding that MRI, particularly advanced techniques like SWI and FLAIR, outperforms CT in detecting subtle and chronic brain injuries in TBI cases. Existing literature has long highlighted MRI’s superior sensitivity to diffuse axonal injury, microbleeds, and other parenchymal abnormalities [14], aligning with studies such as Wu et al. [11] and Sigmund et al. [10], which demonstrated MRI's enhanced ability to detect subarachnoid hemorrhage and distinguish between different severities of brain injury. Previous meta-analyses and reviews, including studies from researchers like Shenton et al. [5], corroborate this, showing that MRI, with its capacity to image soft tissues and assess white matter integrity, is more effective for long-term injury assessment and prognosis in TBI patients. The addition of proton MRS, as discussed in Garnett et al. [12], further extends the understanding of how MRI can detect cellular damage not visible on CT, which is critical for identifying potential delayed neurological decline.

However, while MRI’s superiority for detailed imaging is well-established, this review also reinforces the indispensable role of CT in acute trauma settings. The rapid availability of CT scans and their ability to quickly detect acute hemorrhages and fractures make them a vital first-line imaging modality in emergency care, which remains consistent with the broader clinical guidelines and literature. Studies like McMahon et al. [9] further support the current clinical practice of utilizing CT for immediate assessment, especially when combined with biomarkers like GFAP-BDP to reduce unnecessary scans and improve diagnostic accuracy [15]. Thus, the findings of this review largely support the existing consensus in the field while offering insights into how combined diagnostic strategies may enhance patient outcomes in TBI management.

One of the key strengths of this review lies in its comprehensive search strategy, which adhered to PRISMA guidelines and ensured the inclusion of high-quality studies across a range of TBI severity levels, from mild to severe. The review draws on studies that utilized both advanced MRI techniques and standard CT, offering a well-rounded comparison of their diagnostic capabilities. Additionally, the inclusion of various imaging modalities and biomarkers, such as GFAP-BDP, provides valuable insights into how these tools complement each other in clinical practice. However, there are notable limitations. The heterogeneity of the included studies, particularly in terms of population size, study design, and imaging protocols, posed challenges for direct comparisons. Furthermore, the exclusion of non-English studies may have resulted in the omission of relevant research from other regions. Finally, the absence of a meta-analysis due to the variability in outcome measures limits the ability to quantitatively synthesize the results. Despite these limitations, the review offers a thorough qualitative synthesis of the available evidence, contributing valuable insights into TBI imaging.

The findings of this review suggest that MRI, particularly advanced modalities such as FLAIR and SWI, should be more frequently integrated into the diagnostic pathways for TBI, especially in cases where CT scans may fail to detect subtle injuries, such as diffuse axonal injury or microbleeds. In particular, pediatric and mild-to-moderate TBI patients, who often present with normal CT scans but later develop cognitive or motor impairments, could benefit from an early MRI evaluation. Incorporating MRI into standard follow-up protocols could improve the identification of long-term damage that CT might miss, thereby facilitating early intervention and more personalized treatment strategies. Moreover, the use of biomarkers like GFAP-BDP in conjunction with CT and MRI could offer a more streamlined approach to diagnosing intracranial injuries, potentially reducing the reliance on repeated CT scans and minimizing radiation exposure [16].

In emergency care settings, CT remains indispensable due to its speed and ability to rapidly detect life-threatening conditions like hemorrhage or skull fractures [17]. However, the findings emphasize that MRI should not be overlooked, particularly in stable patients who require a more comprehensive evaluation. In trauma centers with the appropriate resources, early MRI use could enhance diagnostic accuracy, especially for patients showing persistent symptoms despite normal CT results [18-20]. The review also highlights the need for clearer clinical guidelines on the appropriate use of MRI versus CT in different TBI severity and stages, ensuring that both modalities are optimally utilized to improve patient outcomes while balancing costs and resources.

Future research should focus on conducting large-scale, multi-center trials that directly compare the diagnostic performance of MRI and CT in different severities and types of TBI. These studies should aim to standardize imaging protocols and outcome measures, enabling more accurate comparisons across studies. Additionally, further exploration into the combination of imaging modalities with emerging biomarkers like GFAP-BDP could offer new diagnostic pathways, potentially improving early detection and reducing unnecessary imaging [21]. Research should also address the gaps in understanding how MRI, particularly advanced techniques such as MRS, can be used to predict long-term neurological outcomes. Finally, investigating the cost-effectiveness and practical implementation of routine MRI use in clinical practice, particularly in resource-limited settings, will be critical for optimizing TBI management on a global scale.

## Conclusions

This systematic review highlights the complementary roles of MRI and CT in the diagnosis and management of TBI. While CT remains essential in acute care settings due to its speed and ability to detect life-threatening conditions, MRI, particularly with advanced techniques like FLAIR and SWI, offers superior sensitivity in identifying subtle injuries and predicting long-term outcomes. The integration of MRI into routine diagnostic pathways, especially for patients with mild or moderate TBI, could lead to more comprehensive assessments and tailored treatment plans. Additionally, the use of emerging biomarkers like GFAP-BDP alongside imaging modalities may further enhance diagnostic accuracy and reduce unnecessary radiation exposure. The take-home message for clinicians is that a combined approach leveraging both CT and MRI will provide the most thorough evaluation of TBI, improving patient outcomes, while future research should continue to explore these modalities' synergistic potential.
